# Virtual navigation strategies from childhood to senescence: evidence for changes across the life span

**DOI:** 10.3389/fnagi.2012.00028

**Published:** 2012-11-15

**Authors:** Veronique D. Bohbot, Sam McKenzie, Kyoko Konishi, Celine Fouquet, Vanessa Kurdi, Russel Schachar, Michel Boivin, Philippe Robaey

**Affiliations:** ^1^Department of Psychiatry, Douglas Institute, McGill UniversityVerdun, QC, Canada; ^2^Hospital for Sick ChildrenToronto, ON, Canada; ^3^Deptartment of Psychology, Université de LavalQuebec, QC, Canada; ^4^Department of Psychiatry, Ste-Justine Research CenterMontreal, QC, Canada

**Keywords:** hippocampus, caudate nucleus, navigational strategy, spatial memory, twins, children, aging

## Abstract

This study sought to investigate navigational strategies across the life span, by testing 8-years old children to 80-years old healthy older adults on the 4 on 8 virtual maze (4/8VM). The 4/8VM was previously developed to assess spontaneous navigational strategies, i.e., hippocampal-dependent spatial strategies (navigation by memorizing relationships between landmarks) versus caudate nucleus-dependent response strategies (memorizing a series of left and right turns from a given starting position). With the 4/8VM, we previously demonstrated greater fMRI activity and gray matter in the hippocampus of spatial learners relative to response learners. A sample of 599 healthy participants was tested in the current study. Results showed that 84.4% of children, 46.3% of young adults, and 39.3% of older adults spontaneously used spatial strategies (*p* < 0.0001). Our results suggest that while children predominantly use spatial strategies, the proportion of participants using spatial strategies decreases across the life span, in favor of response strategies. Factors promoting response strategies include repetition, reward and stress. Since response strategies can result from successful repetition of a behavioral pattern, we propose that the increase in response strategies is a biological adaptive mechanism that allows for the automatization of behavior such as walking in order to free up hippocampal-dependent resources. However, the down-side of this shift from spatial to response strategies occurs if people stop building novel relationships, which occurs with repetition and routine, and thereby stop stimulating their hippocampus. Reduced fMRI activity and gray matter in the hippocampus were shown to correlate with cognitive deficits in normal aging. Therefore, these results have important implications regarding factors involved in healthy and successful aging.

## Introduction

The human brain changes across the entire life span. Throughout childhood there are changes in the function and size of numerous brain structures which correlate with increased performance on tasks that are dependent upon these regions (Casey et al., 2002; Thomas et al., 2004; Menon et al., 2005). In contrast, decreases in memory and executive function have been observed with normal aging. These deficits have been associated with decreases in the volume of the hippocampus (Lupien et al., 1998; Small et al., 2002; Raz et al., 2004; Moffat et al., 2006) and frontal cortex (Raz et al., 1997; Grady and Craik, 2000; Cabeza, 2002). Despite known neural changes that happen during development and aging, very few human studies have examined the corresponding changes in behavior across the entire lifespan.

Navigation is often used as a model for learning because it is possible to dissociate different learning strategies which depend upon distinct memory systems. Many lines of research in rodents and humans have demonstrated that the hippocampus is required when one must learn the spatial relationships between multiple landmarks in the environment, i.e., when forming a cognitive map of the relationships between environmental landmarks (O'Keefe and Nadel, 1978; Packard et al., 1989; Bohbot et al., 2004). On the other hand, when stimulus-response associations must be made, i.e., by learning a series of specific movements from a given start position or stimulus, the striatum, formed of the caudate nucleus, putamen and nucleus accumbens, is necessary. Under certain experimental conditions, recruitment of the hippocampus has actually been shown to interfere with this form of learning (Packard et al., 1989; McDonald and White, 1993; Hartley et al., 2003). In young adult humans, the spontaneous use of a response strategy during virtual navigation has been associated with increased activity and gray matter of the caudate nucleus portion of the striatum, while the use of a spatial strategy has been related to increased activity and gray matter in the hippocampus (Iaria et al., 2003; Bohbot et al., 2007). Interestingly, a negative correlation between the gray matter of the caudate nucleus and hippocampus was observed (Bohbot et al., 2007), a finding that adds to the growing body of literature describing the fact that only one of the two structures is used at any given time, in a manner that appears competitive (Packard et al., 1989; McDonald and White, 1993; Gold, 2004).

Studies in rodents and humans have suggested that memory deficits in older adults are not uniform and may be specific to the decline of particular structures. In a study by Barnes et al. (1980), it was demonstrated that older rats employed a response strategy to a greater extent than younger rats in a T-maze. Similarly, after young and aged rats learn the location of a submerged platform in the Morris Water Maze, aged rats search more readily for a visible platform in a new location showing bias toward response strategies, as opposed to younger rats who ignore the visible platform and continue searching for the submerged platform in the old target location indicating a bias toward spatial strategies (Rapp et al., 1987). Another study (Nicolle et al., 2003) showed that aged mice were able to use a spatial strategy in the Morris Water Maze when forced to, but predominantly used a response strategy when given the choice. Structural and functional imaging studies have shown hippocampal decline in older adults (Jernigan et al., 2001; Raz et al., 2004; Jernigan and Gamst, 2005; Walhovd et al., 2005; Moffat et al., 2007; Head and Isom, 2010) as well as inferior performance when using processes which depend upon the hippocampus, such as spatial memory (Newman and Kaszniak, 2000; Moffat et al., 2006) and episodic memory (Maguire and Frith, 2003; Persson et al., 2006). Etchamendy et al. (2012) showed that human older adults tested on a virtual analog of a rodent radial task were impaired at using spatial relationships to solve the task, while response learning was intact.

A study (Leplow et al., 2003) has addressed the question of which memory system is spontaneously used in children. In this study, all the children over the age of 10 years old used a spatial strategy. However, it is unclear whether the paradigm used was equally sensitive to the two strategies. Other studies which have not tested for response strategies have found that the development of spatial competence emerges between seven and 8 years of age; about the same time that children can abstract spatial relationships to scaled models (Overman et al., 1996). It is unknown whether school-aged children would depend more on the information processing of the hippocampus or that of the caudate nucleus on a task which can be solved equally well using either learning strategy.

In order to assess the relative contribution of different memory systems across the lifespan, we administered a virtual navigation task that can be learned using either a spatial or response strategy to 299 children, 175 young adults, and 125 older adults. Based on previous studies in rodents and preliminary data with humans from our laboratory, we predicted that children would predominantly use spatial strategies and that response learning strategies would be increasingly used with age.

## Methods

### Participants

Children participants were taken from a sample of 299 eight-years-old twins (monozygotic and dizygotic). Ninety-five young adults who took part in four ongoing studies were added to the sample of 80 young participants tested in two previously published studies (Iaria et al., 2003; Etchamendy et al., 2007) in which the same paradigm was used. Only data from the behavioral studies were used for the current study. In total, 175 young adults (84 men, 91 women, mean age: 25.6 ± 4.6 years, age range: 19–40) were tested. Participants were recruited through word of mouth. A sample of 125 older adults (50 men, 75 women, mean age: 66.5 ± 6.6 years, age range: 53–85) were recruited from newspaper and radio ads.

All participants were screened for neurological and psychiatric disorders, including depression. All older adult participants scored above the normative cutoff score for the Mini Mental State Examination (Iverson, 1998). All participants gave written consent to take part in the study. In the case of child participants, written consent was obtained from the parents. The study was approved by the Research Ethics Board at the Douglas Mental Health University Institute and the Sainte-Justine University Hospital Research Center. Participant recruitment and testing was in conformity with the local ethics committee requirements.

### 4 on 8 virtual maze (4/8VM)

#### Adult version

A commercially available computer game (Unreal; Epic Games, Raleigh, NC) was used to create the virtual environments. The virtual tasks were presented on a 17′ computer screen. Before testing, the participants spent a few minutes moving in a virtual room that was different from the experimental environment to practice the motor aspects of the task. When the participants were comfortable using the keypad, the experimenter gave the instructions, and the experiment started.

**Figure 1:**
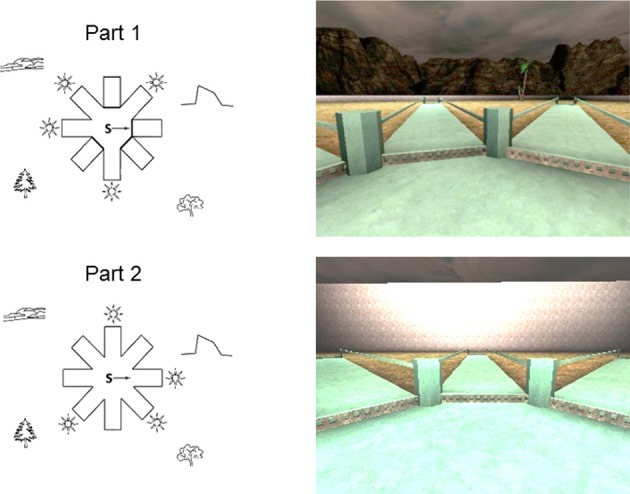
**Schematic drawings (left) and first person views (right) of the adult 4/8VM environment**.

The 4/8VM is composed of an eight-arm radial maze with a central starting location (Figure 1). The maze is surrounded by a landscape (mountains and sunset), two trees, and a short wall located between the landscape and the trees. At the end of each arm are stairs that lead to a pit where, in some of the arms, an object can be picked up. The location of the target objects cannot be seen from the center of the maze. Landmarks in the environment were not located directly in front of the target pathways, thereby avoiding the use of a beacon strategy. The participants used a keypad to move forward, left, and right within the environment and were instructed to not use the backwards key. Participants always started a trial at the center of the radial maze facing the same direction. The 4/8VM consisted of five trials, all of which are composed of two parts. In Part 1 of each trial, four arms were blocked with barriers and the remaining four arms were accessible and contain objects. In Part 2, all arms were accessible and the objects were located in the four arms that were previously blocked in Part 1. The participants were asked to retrieve all objects found in the accessible arms in Part 1 and to remember which arms they visited. In Part 2, they were asked to avoid the arms they previously visited in order to find the objects. Errors consisted of entering into an arm that did not contain an object or revisiting an arm. A trial was completed after all four objects were picked up. Among the five trials, there were three types of trials: type A, B, and C. In Part 1 of trial type A, arms 1, 3, 4, and 6 were accessible and contained objects; in Part 2, the four objects were located at the end of the four previously blocked arms (i.e., arms 2, 5, 7, and 8). In trial type B, a different sequence of accessible arms were used. In Part 1, arms 2, 3, 7, and 8 were accessible, and in Part 2 the objects were located at the end of arms 1, 4, 5, and 6. Trial type C was a probe trial. Part 1 of the probe was identical to part 1 of trial type A. In Part 2, however, the walls around the radial maze were raised to conceal the landscape, and the trees were removed so that no landmarks were visible. During part 2 of the probe trial, all of the arms contained an object. The probe trial was used to distinguish whether participants used a spatial or response strategy to learn the task. If participants were using a spatial strategy in which the landmarks present in the environment were relevant to perform the task, removing the landscape and landmarks should result in an increase in errors. In contrast, if participants were using a response strategy, no increase in errors should occur during the probe trial, since participants would remember a pattern or series of turns in relation to a starting position without relying on the landmarks. To reach criterion and to be allowed to take part in the probe trial, participants were required to make no errors on Part 2 in one of the trials before the probe. The learning criterion was set to ensure that participants were able to learn the task before performing the probe trial which evaluates how they learned the task. Participants were presented with sequences in the order of trial type ABACA. For the young and older adults, data from several studies were combined in order to obtain a larger sample size. Although there were design differences for each of the studies, the portion of the 4/8VM used in the current paper was identical in all studies. In one study, 74 participants received the ABACA sequence whereas in another 50, 13 trials were administered after the ABACA sequence for a total of 18 trials. In the current paper only the ABACA sequence was considered. Participants who were not able to complete a trial without error within the first three trials were given up to two extra trials in order to reach criterion. The extra trials were trial type A.

As previously described (Iaria et al., 2003; Bohbot et al., 2007, 2011; Banner et al., 2011; Andersen et al., 2012), the participants were debriefed at the end of the experiment. They were asked to report how they solved the task from the beginning to the end of the experiment. Participants were classified based on their spontaneous navigational strategy. Participants were categorized as using a response strategy if they initially learned the task by associating the arms with numbers or letters, or by counting the arms (clockwise or counterclockwise) from a single starting point. If they initially used two or more landmarks and did not mention a response strategy, they were categorized as using a spatial strategy. Errors on the probe trial were used to confirm navigational strategies in an objective fashion, where spatial learners were expected to make more errors than response learners. A recent eye tracking paper further validated verbal reports as a method of categorization (Andersen et al., 2012). The study showed that spatial learners spend significantly more time looking at landmarks than response learners.

#### Children's version

**Figure 2:**
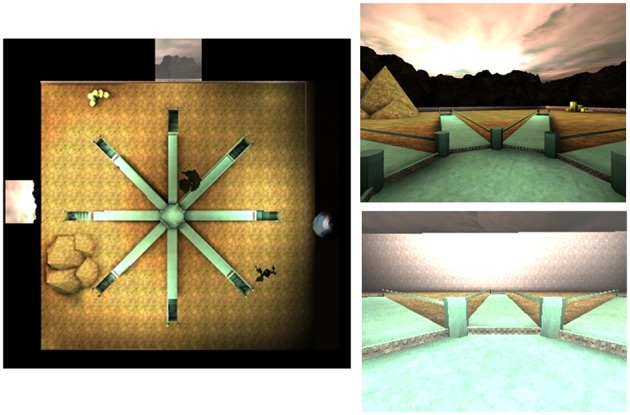
**Top down (left) and first person (right) views of the children 4/8VM environment**.

The 4/8VM described above was also used to test children, however the following modifications were necessary in order to obtain a valid test measuring navigational strategies. The environment contained the same eight-arm radial arm maze surrounded by a landscape (mountains and sunset) and two trees, however, additional landmarks were added: a planet, a pyramid, and a pile of boxes. In addition, instead of presenting Part 1 and Part 2 as we did in one trial of the adult version, one trial in the children's version only consisted of Part 2 where all eight arms are accessible and four objects had to be found. Furthermore, only two types of trials were used for the children: trial types A and C (Figure 2). This modification was made gradually throughout 1.5 years of pilot studies in order to make the test more easily comprehensible and feasible to 8-years old children. In the children 4/8VM, participants were asked to retrieve all objects from the target arms out of the eight open arms. A trial was completed after all four objects were picked up. The target arms were the same arms as those used in the adult version (2, 5, 7, and 8). Due to time constraints, participants were given at most either 10 (version 1, *N* = 51) or 13 trials (version 2, *N* = 209) to learn the target arms (2, 5, 7, and 8) to criteria. As in the adult version, at the beginning of each trial, participants started in the center of the maze facing the same direction. To reach criteria, participants were required to have completed three out of four trials without error. Participants were therefore required to complete a minimum of three trials. For the participants who reached criteria, a second stage of the task, the probe trial (type C), was presented. In the probe trial, similar to the adult version, walls were raised to conceal the landscape and all landmarks were removed. An object was present in every arm. As in the adult version of this task, the purpose of the probe trial was to distinguish the participants who relied on landmarks (i.e., used a spatial strategy) from participants who learned the pattern of target arms irrespective of landmarks (i.e., used a response strategy).

After participants completed the virtual radial arm maze, they were asked how they found each of the objects, following a similar procedure as in adults. Participants were categorized as using a response strategy when they mentioned numbering or counting arms from a given start position, in order to find all the objects (i.e., “I went down the arm directly ahead, the one next to it, then skipped two arms to the right, then skipped one arm”). On the other hand, if participants mentioned at least two landmarks and did not mention using a pattern (i.e., “One was beside the pyramid, one on each side of the tree and one next to the Earth”), they were categorized as using a spatial strategy. The self-reports from the children were less detailed than those of the young and older adults. In order to prevent misclassifications, verbal reports that were ambiguous (e.g., when participants could only report a strategy to find one or two of the goal arms) or did not describe a strategy (e.g., “I just remembered where the arms were”) were excluded. This method of categorization proved to be effective, as we were able to classify 86.48% of our participants.

Errors on the probe trial were used to confirm navigational strategies in an objective fashion. Again, participants using a spatial strategy were expected to make more errors that those using a response strategy, since they relied more on landmarks to find the objects.

In summary, as in the adult study, children were required to remember the position of four objects located in four of the eight arms during acquisition of the task and were required to reach criterion before the administration of the probe trial. The probe trial was used to assess navigational strategy and involved having to retrieve four objects in four of the eight arms in an environment devoid of landmarks and with a hidden landscape.

### Analysis

All analyses were done using SPSS version 15.0 and Microsoft Office Excel 2003. For all participants, task performance was measured by analyzing the total number of errors made when participants had to remember which of the eight open arms contained objects (i.e., Part 2 of each trial). Part 1 errors were not taken into account because errors in a four-arm environment do not provide a sensitive measure of learning and memory for adults and because Part 1 was not administered to children.

During the probe trial, most of the participants in all age groups had the impulse to look around before making their selection and lost their initial heading. Consequently, the pattern of visited arms was used to score errors on the probe trial instead of using the actual arms in absolute space. This was assessed by rotating the pattern of visited arms until we obtained the best match. This method allowed us to distinguish individuals who had learned the pattern of arms (i.e., the response learners) from those who had used the spatial strategy. Specifically, we calculated a probe error term by considering what the number of errors would be if the goal arms were rotated to new positions around the radial arm maze. For example, the goal arms in absolute space were 2, 5, 7, and 8. If a participant initially turned 12.5° clockwise (one arm) and followed the learned relationships between the goal arms thereby making zero errors, they would enter arms 3, 6, 8, and 1, which, if errors were considered in absolute space, would result in three errors. The goal arms were rotated seven times for each possible initial shift in point of view. The best probe error term was then used for further data analysis for all participants. Based on all the possible combinations a participant can make, there is an 8.6% chance of getting two errors randomly, 11.4% chance of getting zero errors randomly, and 80% chance of making one error. Therefore, the mean rotational errors someone can make if they choose randomly is one. Verbal reports were scored by two independent raters showing a 99% agreement in the assessment of strategies for older adults, 93% for young adults and 93% for children.

## Results

### Performance

#### Children

Of these 299 children tested on the virtual radial arm maze, 281 had verbal report and we were able to assess spontaneous strategy in 243 children in total. Of the 299 original participants, 14 children were given the possibility to make 16 choices per trial instead of eight choices. Their verbal reports were analyzed but, to avoid any bias, their performance on the acquisition of the task was not included in the analysis. Among the remaining 285 children tested on the same version of the task (eight choices maximum per trial), 25 participants were excluded due to nausea (*N* = 7), failure to cooperate (*N* = 10), experimental error in administering the task (*N* = 1), and failure to complete the task within the allotted time (*N* = 7). The final sample used for analysis (*N* = 260) consisted of 134 boys and 126 girls and the average age for boys and girls combined was 8.43 ± 0.11 years old.

In total, of the 260 children who completed the study, 205 (78.8%) reached criteria, and 199 completed the probe; six participants who reached criteria did not complete the probe due to time constraints. Children made an average of 6.36 errors (*SD* = 3.53) on the first trial, 3.83 errors (*SD* = 1.79) on the second trial, and 3.05 errors (*SD* = 2.08) on the third trial. Of the 299 children tested, we were able to classify the spontaneous strategies of 243 participants. Of the 243 children, 205 used a spatial strategy (84.4%) and only 38 (15.6%) reported using a response strategy. Children's learning strategy had a profound effect on performance during the probe trial, confirming that our assessment of navigational strategy is consistent with errors on the probe trial. Those that reported using a response strategy made significantly fewer probe errors than those who reported using a spatial strategy, (response mean = 0.69, *SD* = 0.59; spatial mean = 0.90, *SD* = 0.65; one-tail independent samples *t*-test, *p* < 0.05), indicating that response learners relied less on environmental landmarks than spatial learners.

Note that among the 260 children included in the analysis, 51 children were given at most 10 trials (version 1) to reach criteria while 209 were given 13 trials (version 2) to reach criteria. The version of the task did not influence the proportion of children who reached criteria (80.4% in version 1 and 78.5% in version 2) or the average number of errors made during the first 3 trials (4.6 ± 1.7 in version 1 and 4.4 ± 1.7 in version 2). The strategy spontaneously used by the children was also not affected by the version of the task. Spatial strategy was used by 93.2% of the children who were tested on version 1 and 82.01% of the children who were tested on version 2 (chi-square, *p* > 0.05). Taken together, these results indicate that the number of trials given to reach criteria did not influence neither the acquisition of the task nor the strategy used to solve it. Children data from both versions were therefore pooled together for the analyses.

#### Young adults

Of the 175 young adults, seven young adults did not meet criteria by the third trial. At the time of testing these participants were not given extra trials to learn the task before the probe, and therefore their probe performance were not considered in the analysis. Of the 175 young adults, error scores on the learning trials were not available for five participants, however, these participants reached criteria and therefore their strategy and probe scores were included in the analysis. In addition, eight participants did not perform the probe trial although criterion was met.

Young adults made an average of 1.54 errors (*SD* = 2.27) on the first trial (Trial type A), 0.45 errors (*SD* = 1.15) on the second trial (Trial type B), and 0.37 errors (*SD* = 0.9) on the third trial (Trial type A). Of the 175 young adults, 81 (46.3%) reported the use of a spatial strategy and 94 (53.7%) reported using a response strategy. These percentages are consistent with previous reports (Iaria et al., 2003; Etchamendy et al., 2007). Strategy predicted the number of errors made on the probe trial (one-tail independent samples *t*-test *t* = −3.308, *p* < 0.001), whereby response learners made fewer probe errors than spatial learners (response mean = 0.18, *SD* = 0.45, spatial mean = 0.47, *SD* = 0.6), confirming once again that our assessment of navigational strategy is consistent with errors on the probe trial.

#### Older adults

From the sample of 125 participants, 13 participants in total were excluded due to nausea (*N* = 4), failure to complete the task within the allotted time (*N* = 5) and failure to comprehend the task (*N* = 4). We were able to assess spontaneous strategy in 112 older adults (46 men, 66 women; mean age: 66.5 ± 6.7 years).

Of the 112 older adults, 32 subjects did not reach criteria within the first three trials and needed extra trials, of those 16 older adults never met criteria and did not do the probe trial. Older adults made an average of 2.92 errors (*SD* = 2.88) on the first trial (Trial type A), 2.37 errors (*SD* = 2.58) on the second trial (Trial type B), 1.82 errors (*SD* = 2.6) on the third trial (Trial type A). Of the 125 older adults tested, we were able to classify the spontaneous strategies of 112 participants. Of these 112 older adults, only 44 (39.3%) reported the use of a spatial strategy and 68 (60.7%) reported the use of a response strategy. Reported strategy predicted the number of probe errors (response mean = 0.37 errors, *SD* = 0.55; spatial mean = 0.72 errors; *SD* = 0.70; one-tail independent samples *t*-test *t* = 2.76, *p* < 0.01), confirming once again that our assessment of navigational strategy is consistent with errors on the probe trial. In the event that giving additional trials could influence navigational strategy, a separate analysis was performed with only the older adult participants who reached criteria in three trials. Results showed that the proportion of spatial and response learners did not differ from the whole group analysis: 62.4% used a response strategy and 37.6% used a spatial strategy. Therefore, giving older adult participants extra trials did not affect their navigational strategy. In support of these findings, giving extra trials to children did not increase their rate of using a response strategy. We further argue that a shift toward response strategies only occurs with over-training, after participants perform the task to criteria.

#### Children, young adults and older adults

When looking at all of the participants, 199 children, 160 young adults, and 96 older adults reached criteria and performed the probe trial. There was a significant difference in performance between children, young adults, and older adults on the first three learning trials [*F*_(2, 540)_ = 298.76; *p* < 0.0001]. During the learning trials, participants had to avoid the arms that they had previously visited in order to retrieve the objects. Young adults made significantly fewer errors than the older adults and both groups performed significantly better than the children (*post-hoc* tests: *p* < 0.001). All participants who met criteria obtained a perfect score in at least one learning trial before the probe. On the last trial, children that reached criteria and did the probe made no errors. Older adults made an average of 0.65 errors (*SD* = 1.89) and young adults made an average of 0.37 errors (*SD* = 0.9) on the last trial before the probe.

### Strategy

#### Children

Among the 199 children who reached criteria and did the probe, we were able to assess spontaneous strategy in 191 participants. Within these 191 children, we found that 83.3% were spatial learners and 16.7% were response learners. These proportions are similar to those found when looking at the 281 participants who had verbal report and no probe score. Strategy did not predict performance on the acquisition of the 4/8VM (before the probe trial). Strategy did not influence the number of errors made during the first three trials (independent samples *t*-test, *p* > 0.05). Similarly, strategy did not affect the number of trials needed to reach criteria (independent samples *t*-test, *p* > 0.05 in both versions of the task). There was no relationship between sex and strategy in the children population (chi-squared, *p* > 0.05).

#### Young adults

As in the children sample, strategy did not predict the number of errors made during the first three trials [*F*_(2, 167)_ = 0.065, *p* > 0.05]. There was no relationship between sex and strategy in the young adult population (chi-square, *p* > 0.05).

#### Older adults

Strategy had no effect on performance as measured by the number of errors made in the first three trials [*F*_(2, 107)_ = 0.28; *p* > 0.05]. Strategy also did not predict the decrease in errors between the first and second A trials (*t* = 0.208; *p* > 0.05). Strategy did not interact with older adults' ability to reach criteria by the third trial (chi-square; *p* > 0.05). There was no relationship between sex and strategy in the older adult population (chi-square, *p* > 0.05).

#### Comparison of the strategies of children, young adults, and older adults

**Figure 3:**
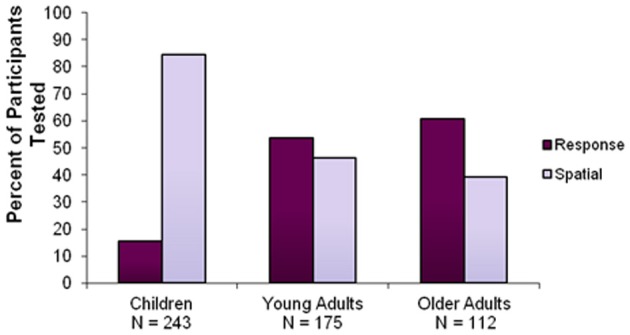
**Percentage of participants using a spatial or response strategy according to age group.** This graph shows that the use of response strategies increases across the life span, at the expense of spatial strategies.

A 3×2 chi-square (children, young adults, older adults × spatial, response) was used to compare the proportion of spatial and response learners across all age groups. The chi-squared analysis revealed a significant interaction [χ^2^ = 94.69, *p* < 0.0001] between age and strategy, with a decrease in the use of a spatial strategy throughout the lifespan: 84.4% of children, 46.3% of young adults, and only 39.3% of older adults reported the use of a spatial strategy (Figure 3). *Post-hoc* analysis revealed a significant difference in the proportion of spatial and response learners between children and young adults [χ^2^ = 68.26, *p* < 0.0001] and children and older adults [χ^2^ = 74.38, *p* < 0.0001]. There was no significant difference in the proportion of spatial and response learners between young and older adults [χ^2^ = 1.361, *p* > 0.05].

**Figure 4:**
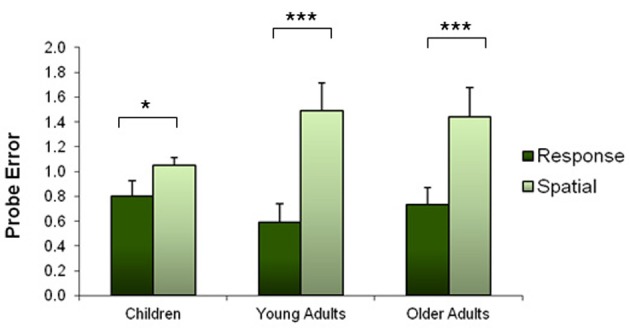
**Normalized mean number of probe errors according to strategy and age group.** This graph shows that probe errors were effective at dissociating spatial and response strategies: spatial learners made more errors on the probe trial than response learners. Error bars represent standard error of the mean. ^*^p < 0.05; ^***^p < 0.001.

The probe trial was successful at discriminating between spatial and response strategies. A 3 × 2 ANOVA (age × strategy) revealed a significant main effect of strategy on probe performance [*F*_(2, 447)_ = 9.96, *p* < 0.001]. As anticipated, there was no interaction between age and strategy because response learners performed better than spatial learners on the probe trial across all ages [*F*_(1, 447)_ = 0.221, *p* = 0.638]. In order for the groups to be comparable, a normalized error term was calculated for each individual such that the average probe error equals one in each group. Figure 4 shows the normalized probe errors according to strategy and age group.

## Discussion

Our findings demonstrate a shift in navigational strategy across the lifespan (Figure 3). We found that 84% of children reported using a spatial strategy, indicating a clear bias compared to the 47% of young adults who reported using the same strategy. We also observed that 39% of older adults used a spatial strategy. Moreover, we found that response learners performed better on the probe than spatial learners in every age group, a finding which extends the results of previous studies carried out in young adults (Iaria et al., 2003; Bohbot et al., 2007) to children and older adults (Figure 4). These results suggest that, across all age groups, the probe trial was effective in discriminating spatial learners who relied on environmental landmarks from response learners who were not as affected by the removal of landmarks.

Young adults performed better than older adults on trials 1, 2, and 3, which is consistent with general navigational deficits that have been observed in normal aging (Marighetto et al., 1999; Driscoll et al., 2005). While some caudate nucleus-dependent deficits have been observed with aging (Barrash, 1994; Wilkniss et al., 1997; Meulenbroek et al., 2004; Raz and Rodrigue, 2006; Head and Isom, 2010), human and animal studies show that these deficits are milder or relatively spared in old age (Vasquez et al., 1983; Grady and Craik, 2000; Churchill et al., 2003). Bach et al. (1999) tested young and old mice on a Barnes circular maze and found that old mice had deficits in spatial memory compared to young mice. The same study also showed that on a cued version of the same task, a version that requires the formation of stimulus-response associations, aged mice performed similar to young mice, demonstrating the sparing of response learning with age. Interestingly, during learning, after the initial random exploration, aged mice adapted a serial search strategy that does not rely on the hippocampus while young mice adapted a spatial search strategy dependent on the hippocampus. Rapp et al. (1997) found similar results in monkeys where older monkeys used a serial search strategy compared to young monkeys. Authors also found that older monkeys were not affected by the removal of external maze cues, demonstrating their lack of reliance on spatial landmarks. Our study translates these results to humans and reveals a more complex picture for cognitive aging. The deficit seen in normal older adults in the current study is paralleled with a shift toward using response strategies, an effect first demonstrated in rats by Barnes et al. (1980). Response strategies are efficient when navigating in an environment where the start and target locations are constant, as in route learning paradigms (Hartley et al., 2003; Iaria et al., 2003; Head and Isom, 2010). In contrast, when the relationship between the start and target position changes and a novel path must be derived, response strategies are inefficient (Hartley et al., 2003; Driscoll et al., 2005). Deriving a novel path from different start and target locations requires knowledge of a cognitive map, making the use of spatial strategies more efficient. The drive toward efficiency may be an important underlying factor behind the shift in strategies with normal aging. With the repetition of a successful behavior, a response strategy emerges, leading to the automatization of behavior or habit formation (Iaria et al., 2003). However, this shift toward response strategies comes at a cost when a novel path must be derived using a cognitive map in order to navigate successfully.

Other lifestyle factors can produce a shift from using spatial strategies to response strategies with aging. For example, stress, as well as addiction related rewards such as nicotine, opiates, psychostimulants, and alcohol have been shown to affect the integrity of the hippocampus. Stress was reported to impair the hippocampus through the actions of glucocorticoids (Sapolsky et al., 1990; Sapolsky, 1994; McEwen and Sapolsky, 1995; Conrad et al., 1996; McKittrick et al., 2000; Kleen et al., 2006) and was shown to have an effect on navigational strategies. Schwabe et al. (2007, 2008, 2010, 2012) found that chronic stress, acute stress, and prenatal stress can increase the use of response strategies in people tested on a navigation task. Taking into consideration the inverse relationship between hippocampus and caudate nucleus gray matter (Bohbot et al., 2007) and the fact that rewards stimulate the caudate nucleus, we can expect the probability of using caudate nucleus-dependent response strategies to be higher in people exhibiting reward-seeking behaviors. Supportive evidence is found in a study showing that rewards lead to increased goal-oriented navigation and decreased free exploration, the latter of which is characteristic of spatial memory, in healthy adults performing a virtual water maze task (Adcock, 2010). These studies demonstrate that stress and reward can promote the use of response strategies.

Children, including both spatial and response learners had a higher number of probe errors than adult spatial and response learners. These data support the finding that children preferentially use their hippocampus to navigate, since high probe errors have been shown to be associated with greater hippocampal involvement (Iaria et al., 2003). Even the children that used caudate nucleus-dependent response strategies over hippocampus-dependent spatial strategies showed higher probe deficits as compared to young adults, suggesting that response learning may also be immature at 8 years of age. As an alternative to the view that the striatum is mature by childhood (Reber, 1992; Maybery and O'Brien-Malone, 1998), we propose that response learning, especially in difficult tasks, may also continue to develop through childhood and adolescence (Casey et al., 2002; Sowell et al., 2002; Thomas et al., 2004). Although children are capable of using patterns to learn, the pattern formation required in the current task that leads to the use of a response strategy, is much more sophisticated compared to a simple “egocentric” strategy, involving a single vector addition from a given position, reported in numerous children studies (Overman et al., 1996; Bremner and Bryant, 1977). As opposed to navigating toward a beacon, successful probe performance in our task requires participants to keep in memory a sequence of movements through virtual space. In other tasks that require more complex sequence learning, children have been shown to be impaired relative to adults and there is increased activation in the caudate nucleus when age is correlated with performance (Thomas et al., 2004). Though children may be capable of using an egocentric strategy from a very young age, even before the emergence of spatial strategies (Lehnung et al., 1998), the complex pattern of stimulus-response associations required in the current task seems to evolve later on.

Unlike previous reports that used tasks for which all adults used a spatial strategy (Bullens et al., 2010), we have shown that in learning situations where young adults are equally likely to adopt a spatial strategy or a response strategy, children are biased toward using spatial strategies. It is of interest that we found spatial strategies dominating in childhood, since these results show a bias toward hippocampal-based learning in the early stages of life, despite the immaturity of the hippocampus (Saitoh et al., 2001; Pine et al., 2002; Mulani et al., 2005; Lavenex et al., 2007). We recently replicated these findings in children using identical testing environments. We showed that 7–9 years old children use spatial strategies in greater proportions than older participants who in this case were older children 10–18 years of age (Lin et al., 2012). The caudate nucleus is a slow learning system that develops habits through repetition across a session (Iaria et al., 2003; Orban et al., 2006), days (Packard and McGaugh, 1996; Barnes et al., 2005), and potentially much longer periods of time depending on the complexity of the task. In childhood, most experiences are new and thus children have a smaller repertoire of habits because they have less experience in the world than adults. We hypothesize that this paucity of repetitive behavior is the reason we found a smaller proportion of children using caudate nucleus-dependant response learning and a greater percentage using hippocampal-based spatial learning. Interestingly, in young preweanling rats, the existence of place, head-direction, and grid cells have been shown even before rat pups begin exploring an environment. This finding demonstrates that the mechanisms necessary for building cognitive maps exist early on in development (Wills et al., 2010).

The traditional view that older adults tend to use response strategies because of an aging process, which negatively affects the hippocampus, suggests a compensatory mechanism for this shift in strategies (Etchamendy et al., 2012). We offer an alternate hypothesis whereby a shift in navigational strategy with time is a consequence of the increased use of the caudate nucleus-based response learning in older adults (Balram et al., 2010). We suggest that it is biologically adaptive for the caudate nucleus to automatize frequently repeated behavioral and cognitive processes such as learning how to walk, in order to free up cognitive resources (Albouy et al., 2008, 2012). This process, however, will result in a bias toward the memory encoding strategies dependent on the caudate nucleus and decrease the need to make novel relationships between multiple stimuli, a process which requires the hippocampus (Eichenbaum et al., 1992). With aging, people often gain expertise in a specific field through professional and personal life experiences. Gaining expertise, however, often involves carrying out processes faster and more efficiently. We argue that repetitive day-to-day behavior decreases the likelihood of experiencing difficult and long processes normally required for learning new things.

Normal aging is accompanied by a decrease in hippocampal volume and functional activity, which is associated with navigational deficits (Driscoll et al., 2005) in spatial memory tasks (Moffat et al., 2006; Antonova et al., 2009; Chen et al., 2010; Head and Isom, 2010). We suggest that decreases in hippocampal volume and activity could be a consequence of increased use of response strategies. In fact, recent studies by Rodgers et al. (2012) and Etchamendy et al. (2012) showed that a larger proportion of older adults use response strategies compared to young adults. When middle age healthy participants (mean age = 43.0 ± 5.9 years) were tested as a control group for patients with damage to the hippocampus in Bohbot et al. (2004), 85% used a response strategy on the 4/8VM. Although in the current study we did not find a direct difference in the proportion of spatial and response learners between young and older adults this may in part be due to stringent screening for various disorders, more common in older adults than in young adults. In Bohbot et al. (2004) healthy participants were recruited by word of mouth and were often spouses of the brain-damaged patients because they were balanced for age, education and socio-economic status. On the other hand, participants recruited for studies comparing young and older adults have to be screened for numerous factors. This screening process results in a greater exclusion rate in older adults than in young adults making the older adult population a very healthy sample, free of neurological, psychiatric, metabolic (e.g., heart attack, cholesterol, diabetes), and chronic diseases (e.g., cancer). As previously mentioned, the automatization of behavior may be a biologically adaptive mechanism that permits us to free up resources, such as hippocampal function. However, less hippocampal engagement may lead to decreased hippocampal gray matter and volume, which is associated with cognitive deficits in normal aging and is a risk factor for developing dementia (Lupien et al., 1998; Tisserand et al., 2004). This suggestion fits with the finding that years of education lowers the risk of developing dementia (Ravaglia et al., 2002; Karp et al., 2004; Caamano-Isorna et al., 2006), presumably because the hippocampus was involved in making novel relationships for a longer period of time while people were learning new information. Thus, having more years of education may play a role in delaying the shift toward response strategies. Similarly, James et al. (2011) found that people with a larger life space, measured by more movement through the Chicago area during their daily activities, have a decreased risk of developing dementia. Longitudinal studies of successful aging have also highlighted the importance of participating in everyday activities which require the learning of novel information (Hultsch et al., 1999), a finding which gives credence to the “use it or lose it” hypothesis presented here. In support of these studies, our mouse imaging study showed an inverse relationship between gray matter in the hippocampus and striatum when mice trained on a spatial memory version of the water maze were contrasted to mice trained on the response memory version in absence of the possibility of using spatial strategies because landmarks were hidden with a curtain (Lerch et al., 2011). In sum, the use of spatial strategies may have protective effects on the hippocampus.

In conclusion, with age, people who use response strategies to a great extent in their everyday life may be more at risk of developing cognitive deficits in normal aging and dementia (Dossa et al., 2010), through increased caudate-dependent learning and decreased hippocampal-dependent processing. An emphasis on cognitive mapping may increase the functioning and gray matter of the hippocampus, both of which increase the probability of healthy and successful aging. Thus, reversing the shift toward response strategies that comes with age with spatial memory training may be an effective method of prevention against cognitive decline and dementia.

### Conflict of interest statement

The authors declare that the research was conducted in the absence of any commercial or financial relationships that could be construed as a potential conflict of interest.
